# Hierarchically Reinforced PDMS–Silica Coatings for Durable Superhydrophobicity

**DOI:** 10.1021/acsomega.6c01949

**Published:** 2026-07-03

**Authors:** Agneyarka Mohapatra, Somnath Ghosh

**Affiliations:** Department of Chemical Engineering, 28817 Indian Institute of Technology Delhi, New Delhi 110016, India

## Abstract

Robust, self-cleaning surfaces were fabricated by embedding dual-sized silica nanoparticles (SiO_2_, 100 and 500 nm SNPs) into a poly­(dimethylsiloxane) (PDMS) matrix and coating the resulting composite onto a solid substrate. The incorporation of nanoparticles generated a hierarchical surface texture that promoted the emergence of superhydrophobicity. Scanning electron microscope (SEM) analysis confirmed the successful embedding and uniform distribution of the SNPs within the PDMS matrix. To further enhance the mechanical stability of the coating, glass beads (GBs) of different sizes were introduced, providing additional microscale topography and reinforcing the coating against physical damage. This multiscale structuring amplified the water repellency effect. Scratch tests showed that GBs reinforced coatings maintained higher hydrophobicity than their bead-free counterparts. The optimized coating exhibited a water contact angle exceeding 159° and a sliding angle of around 15°, indicating considerable water repellency. Overall, this study demonstrates the role of hierarchical architecture and mechanical reinforcement in developing durable superhydrophobic surfaces suitable for practical applications.

## Graphical abstract

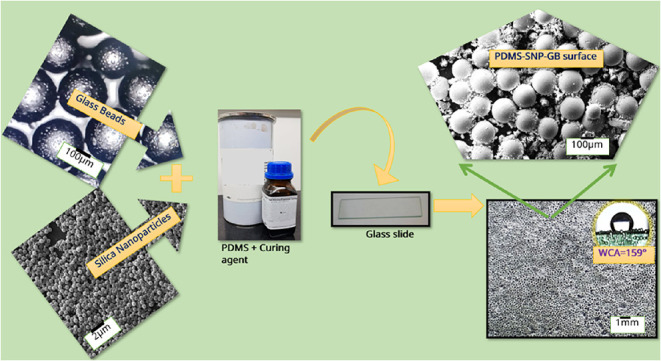

## Introduction

Superhydrophobic (SH) surfaces have emerged as a rapidly growing area of research due to their ability to repel water, resist contamination, and maintain functionality under diverse operating conditions.^1^ Such surfaces are typically characterized by an equilibrium water contact angle (WCA) greater than 150° and a low water sliding angle (WSA) around 10°.^2,3^ These properties provide the surfaces with remarkable self-cleaning capabilities, enabling droplets to roll off and remove dust or microorganisms in the process.^4^ Owing to these properties, SH coatings have been widely explored for applications including self-cleaning windows,^5^ solar panels,^6^ protective textiles,^7^ anticorrosion layers for industrial machinery^8^ and marine vessels,^9^ as well as anti-icing and antifogging surfaces in transportation systems.^10^ With their versatility, SH surfaces are transforming the industries by providing sustainable, efficient, and durable solutions. In addition to macroscale applications, these surfaces show considerable promise at the micro- and nanoscale, offering enhanced resistance to biofouling and enabling precise control over liquid behavior.^11,12^

The extreme water repellency of SH surfaces arises from the combined effects of hierarchical surface roughness and low surface energy material.^13^ This design strategy mimics natural systems like the lotus leaf,^14^ where micro- and nanoscale asperities promote the entrapment of air pockets at the solid–liquid interface, preventing water droplets from adhering and allowing them to roll off effortlessly.^15^ The addition of low surface energy materials further minimizes surface wettability by reducing solid–liquid interactions.^16^ The transition between these regimes can be tuned by carefully tailoring both the topography and the chemistry of the material, offering a powerful strategy to engineer SH coatings with desired performance.^17–19^

Among the material systems investigated, composites based on silica nanoparticles (SiO_2_ NPs, SNPs)^20^ and poly­(dimethylsiloxane) (PDMS) represent one of the most cost-effective and scalable routes for fabricating SH surfaces.^21^ SNPs provide tunable roughness through controlled deposition,^22^ while PDMS provides intrinsic hydrophobicity, chemical stability, and strong adhesion to diverse substrates.^23^ The incorporation of SNPs into a PDMS matrix not only enhances multiscale surface roughness but also stabilizes trapped air layers beneath water droplets, enhancing water repellency.^24^

Conventional strategies to further suppress interfacial interactions often rely on surface modifications. Hydrophobic compounds, such as trichloromethylsilane^25^ or octadecyltrichlorosilane,^26^ are often employed to further reduce surface energy and impart long-lasting hydrophobicity.^27^ However, these treatments frequently involve the release of volatile organic compounds (VOCs), which can cause respiratory irritation and raise environmental concerns.^28,29^ As a result, recent efforts have been focused on environmentally benign strategies, including tailored mixing, curing, and layering of SNP–PDMS composites,^30^ to achieve durable superhydrophobicity and strong interfacial bonding without relying on hazardous chemicals. Such approaches provide both sustainability and scalability, making them suitable for industrial adoption.^31,32^

Despite these advantages, PDMS–SNP coatings often suffer from limited mechanical durability. The inherently soft PDMS matrix is prone to deformation and cracking under stress, while nanoparticle-only coatings typically exhibit poor abrasion resistance. These mechanical limitations often lead to the degradation of superhydrophobic performance. Introducing rigid microscale reinforcements into composite architecture offers an effective strategy to address these challenges. Glass Beads (GBs), in particular, can serve as mechanically robust fillers that distribute applied stresses and suppress crack propagation.^33^The spherical shape of the GBs minimizes stress concentrations while their embedding within the PDMS matrix enhances adhesion to the substrate and suppresses delamination. It also provides additional microscale roughness that complements the nanoscale features of SiO_2_ NPs.

The dual-sized SiO_2_ (100/500 nm) builds on common hierarchical strategies,^39^ but the true novelty lies in GB integration for mechanical reinforcement and scratch resistance, absent in reviewed PDMS/silica works in Table 1, enabling superior physical durability over chemical/UV-focused priors. This multiscale approach (SNPs + micro-GBs) amplifies practicality for hard surfaces with comparable repellence but enhanced longevity.

**Table 1:** Comparative Table of Relevant Previous Reports on Superhydrophobicity with PDMS/SNPs

study	substrate	contact angles (°)		coating method	durability test	key performance parameters
Zhao et al. (2018)^34^	glass	WCA = 169.8	spray (methylated silica aerogel/PDMS + Qsil 216 adhesive)		abrasion 4+ sandpaper cycles, 350 °C/4h, 6 months ambient	thermal/mech stability; semitransparent
		WSA > 4				
Cortese et al., (2008)^35^	PDMS elastomer films	WCA = 160	hierarchical surface roughness via replication/mechanical processing of PDMS surfaces		mechanical stability of rough PDMS	superhydrophobicity due to hierarchical-scale roughness of PDMS
		WSA < 6				
Rahmawan et al., (2010)^36^	Si wafer, glass (DLC-coated)	WCA = 160	wrinkling + dual-scale DLC (diamond-like carbon) fabrication; PDMS not main binder		mechanical robustness of wrinkled DLC; adhesion to substrate	DLC-based dual-scale hierarchical roughness yields superhydrophobicity; mechanically stable wrinkled structures
		WSA = 7				
Zhang et al., (2025)^37^	rigid substrates (e.g., metal, glass)	WCA = 162	PDMS matrix + quartz@SiO2 filler coating; enhanced cross-linking for wear resistance		wear resistance (Taber abrasion/sandpaper), mechanical robustness tests	High-durability PDMS/quartz@SiO2 superhydrophobic coating
		WSA = 5				
*This work*	*solid substrates (glass/steel/wood/ceramic implied)*	WCA = 159	*embedding dual-sized SiO2 (100 and 500 nm) into PDMS + coating; glass beads (GBs) added for reinforcement*		*scratch tests (GB-reinforced > bead-free); mechanical stability emphasized*	*hierarchical nano + micro texture; GBs enhance scratch resistance and durability*
		WSA < 15				
Gong et al.(2020)^38^	glass, plastic, paper	WCA = 156.4	spray coating(PDMS + hydrophilic/hydrophobic SiO_2_)		UV 72h, tape peel 250 cycles, sand abrasion 50g, water impact 5000 drops, pH 1–14 (72h)	self-cleaning; ∼ 40% transmittance on glass
		WSA < 5				
Ke et al., (2011)^39^	not specified (rigid surfaces)	WCA = 156	Sol–gel + dip/spray deposition of silica micronanoparticles in PDMS matrix		mechanical robustness emphasized; abrasion test on sandpaper (qualitative)	mechanically robust superhydrophobic surface using hierarchical silica in PDMS
		WSA = 4.5				
Majeed et al.(2024)^40^	glass	WCA = 156	Dip coating (SiO_2_ + silicon sealant/PDMS)		not performed	self-cleaning on various substrates
		WSA < 10				
Dheeraj et al.(2025)^41^	glass	WCA = 154–161	Dip coating (multilayers)		not performed	>90% transmittance; hierarchical roughness
		WSA < 10				

In this work, we engineer a mechanically reinforced, hierarchical PDMS–silica interface by integrating dual-sized silica nanoparticles (100 and 500 nm) and glass beads within a PDMS matrix. SNPs were synthesized using the well-established Stöber process and systematically incorporated at varying loadings to identify the optimal composition for maximizing water repellency. An equal mixture of both SNP sizes was employed to generate a multiscale surface architecture. Subsequently, GBs were introduced to provide microscale topography and mechanical reinforcement. GBs primarily contributed to stress distribution and abrasion resistance, whereas SNPs embedded within the PDMS matrix generated hierarchical roughness essential for sustaining superhydrophobic behavior. The resulting composite coating exhibits a robust interface that combines high water repellency with improved mechanical durability, demonstrating a scalable strategy for designing durable superhydrophobic surfaces.

## Materials and Methods

### Materials

PDMS, along with curing agent Slygard 184, was purchased from Dow Chemicals Co. Ltd., tetraethyl orthosilicate (TEOS) precursor, and ammonium hydroxide (NH_4_OH) (28–30% NH_3_ basis) used as a catalyst, were purchased from Sigma-Aldrich. Ethanol (EtOH) (99.9%) solvent for washing grade is purchased from Chengshu Hongsheng Fine Chemical Co. Ltd. EtOH for synthesis is purchased from Fisher Chemical, and silica GBs (0.25–2 mm in diameter) are obtained from local vendors. All chemicals were used as received. Deionised water (DI) was obtained from Organo Biotech Laboratories Pvt. Ltd.

### Synthesis of Silica Nanoparticles

Silica nanoparticles (SNPs) of two distinct sizes (100 and 500 nm) were synthesized using the well-established Stober’s process.^42^ In a typical synthesis of 100 nm SNPs, ethanol (EtOH) and deionized (DI) water were mixed in a 4:1 volume ratio (80 mL EtOH and 20 mL DI water) in a clean glass beaker. And stirred for 15 min prior to TEOS addition. Ammonium hydroxide­(aq) (NH_4_OH, 1 mL,0.15M) was then added as a catalyst under continuous magnetic stirring. Silica precursor solution TEOS was introduced dropwise (≈1.5 mL) into the reaction mixture and allowed to react for 2 h at room temperature. The resulting colloidal suspension was centrifuged at 9000 rpm, for 5 min to collect the nanoparticles, which were subsequently washed and redispersed in EtOH and DI water three times to remove unreacted TEOS and residual NH_4_OH. Finally, the purified SNPs were stored by dispersing in EtOH. To prepare 500 nm particles, we followed the same protocol and added 4.2 mL of TEOS after adding the catalyst. The resulting particles were purified and stored in EtOH following the same method as mentioned above.

### Fabrication of PDMS-SNPs SH Surface

First, we have verified the enhanced hydrophobic behavior of PDMS-SNPs composite surfaces. PDMS and the curing agent (Sylgard 184) were mixed in a 10:1 weight ratio and stirred until the mixture was homogeneous and bubble free. A thin uniform PDMS film was then deposited onto the glass substrate by spin-coating at 3000 rpm for 60s. Prior to coating, glass slides were cleaned with ethanol, rinsed with deionized water, and dried in a hot-air oven to remove contaminants. The coated slide was then kept in a hot air oven at 80 °C for 5 min for predrying. This allowed the PDMS to cure partially. Silica nanoparticle (SNP) suspensions in ethanol (100 nm, 500 nm, or mixtures thereof at varying weight fractions relative to PDMS) were spray-coated onto the stipulated area on partially cured PDMS surface to ensure uniform coverage. The substrate was subsequently cured in an oven at 80 °C for 4 h.^43^ This enables complete cross-linking and strong adhesion of SNPs to the surface. After curing, the substrate was cooled to room temperature and stored under ambient conditions for further analysis. With this protocol, we prepared three types of composites by varying the weight ratios of PDMS and silica nanoparticles: (a) PDMS with 100 nm SNPs [PDMS-SNP (100)], (b) PDMS with 500 nm SNPs [PDMS-SNP (500)], and (c) PDMS with a mixture of both the particles [PDMS-SNP (mix)]. In all of the particles embedded surfaces, we observed enhanced hydrophobicity. The fabrication process is schematically illustrated in Figure 1.

**Figure 1:**
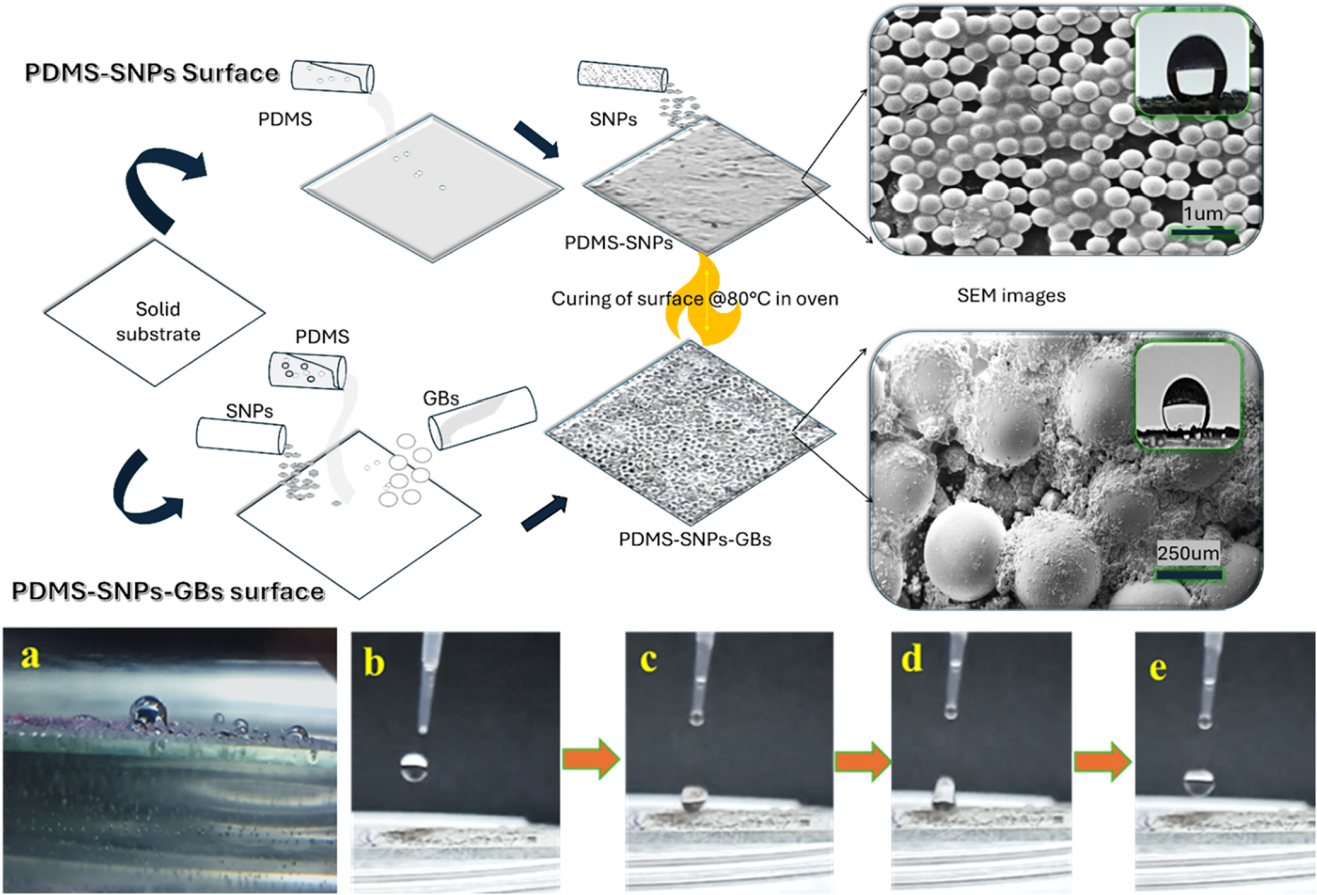
Schematic of the procedure to make the PDMS-SNPs surface and the PDMS-SNPs-GBs surface. Further, a snapshot of a water droplet on the respective surface (inset) indicates that the hydrophobicity was obtained successfully. (a) Snippet of the plastron effect of the coated sample dipped in water. (b–e) Snippets of jumping of a droplet of 2 μL volume of the coated sample.

### Fabrication of PDMS-SNPs-GBs SH Surface

To generate hierarchical micro/nanoscale roughness, micron-sized glass beads (GBs) of five different diameters (250, 500, 1000, 1500, and 2000 μm) were evenly distributed over the partially cured PDMS layer prior to complete cross-linking. Each size bead was used to prepare a separate surface. The beads–PDMS substrates were precured at room temperature for 5 min to allow partial hardening, after which silica nanoparticle (SNP) suspensions (100 nm, 500 nm, or equal mixtures of both) were uniformly spray-coated onto the bead-decorated surfaces. For mixed-particle coatings, dual-sized SNP suspensions were applied in the same manner. The samples were then cured in an oven at 80 °C for 30 min to achieve complete PDMS cross-linking and strong adhesion of both beads and nanoparticles. These GBs contribute to the generation of surface topography. A schematic illustration of this process is shown in Figure 1.

### Surface Characterizations

The wettability of the surfaces was evaluated by measuring the equilibrium contact angle (WCA) and sliding angle (WSA) of water droplets. We measured the contact angles by using DSA100 Drop Shape Analyzer (Krüss Scientific Co. Ltd.) with a water drop of ∼2 μL onto the surface.^38^ The WSA was measured by gradually tilting the sample stage until the droplet began to roll off, indicating the sliding threshold. Surface morphology and the uniform distribution of glass beads (GBs) were examined using an optical microscope (Dewinter Optical Inc.). Microstructural details of the surfaces were further characterized by scanning electron microscopy (SEM, ZEISS EVO 50). The silica nanoparticles were also imaged using a TESCAN MAGNA field emission scanning electron microscope (FESEM) to confirm their size and morphology. Elemental or compositional analysis of the surfaces was conducted using Fourier Transform Infrared (FTIR) spectroscopy (Thermo NICOLET - IS-50) scanning over a range of 400–4000 cm^–1^ to identify characteristic functional groups and chemical bonds. Atomic force microscopy (Asylum Research MFP3D-BIO) was used to analyze the roughness on the SH surface. To evaluate the universality of the composite coating, it was applied to a range of substrates, including metal plate, wood, ceramic, and concrete. The mechanical and chemical stability of the coated surfaces was systematically examined. Mechanical durability was assessed through knife scratch tests, tape peeling, and sandpaper abrasion tests mentioned in the Supporting Information. While chemical stability^44^ was evaluated, bringing out the best coated sample upon immersing it in solutions spanning a wide pH range­(1–13 scale), including acidic and basic environments. Thermal stability^45^ was examined by immersing the samples in hot water at a temperature ranging from 20 to 150 °C. Thereafter, we also studied its durability in exposure to 100 °C boiling water for 1.5 h. To show the air trapping mechanism, plastron effect of the coated sample when dipped inside the water bath is shown in Figure 1a and Video (SI-1) along with the jumping of the water droplet shown in Figure 1(b–e) and Video (SI-2) characterizes the water repelling behavior.

## Results and Discussions

### Fourier Transform Infrared Spectroscopy Analysis

We analyzed the chemical structure of the PDMS–SNP composites using FTIR spectroscopy. Figure 2 shows the corresponding FTIR spectrum confirming the presence of characteristic functional groups. We observed a strong band at ∼1100 cm^–1^ corresponding to Si–O–Si asymmetric stretching vibrations from both the siloxane backbone of PDMS and the silica nanoparticles. We also detected Si–CH_3_ bending at 1251 cm^–1^ and C–H stretching at 2951 and 2850 cm^–1^, which we attribute to PDMS methyl groups that enhance hydrophobicity. A broad absorption band near 3420 cm^–1^ revealed residual −OH groups on the SNP surfaces. After embedding the SNPs into PDMS, the intensity of this band decreased, confirming that the PDMS matrix effectively covered the hydroxyl groups, reduced surface polarity, and increased hydrophobicity. The coexistence of characteristic silica and PDMS peaks, along with enhanced Si–O–Si vibrations in the 1100–1200 cm^–1^ range, demonstrates that the SNPs and PDMS form interfacial interactions. The reduced −OH signal further verifies the transformation of the bulk composite hydrophobic. These results show that the PDMS–SNP composites achieve the chemical synergy necessary for robust, durable, and superhydrophobic surfaces.

**Figure 2:**
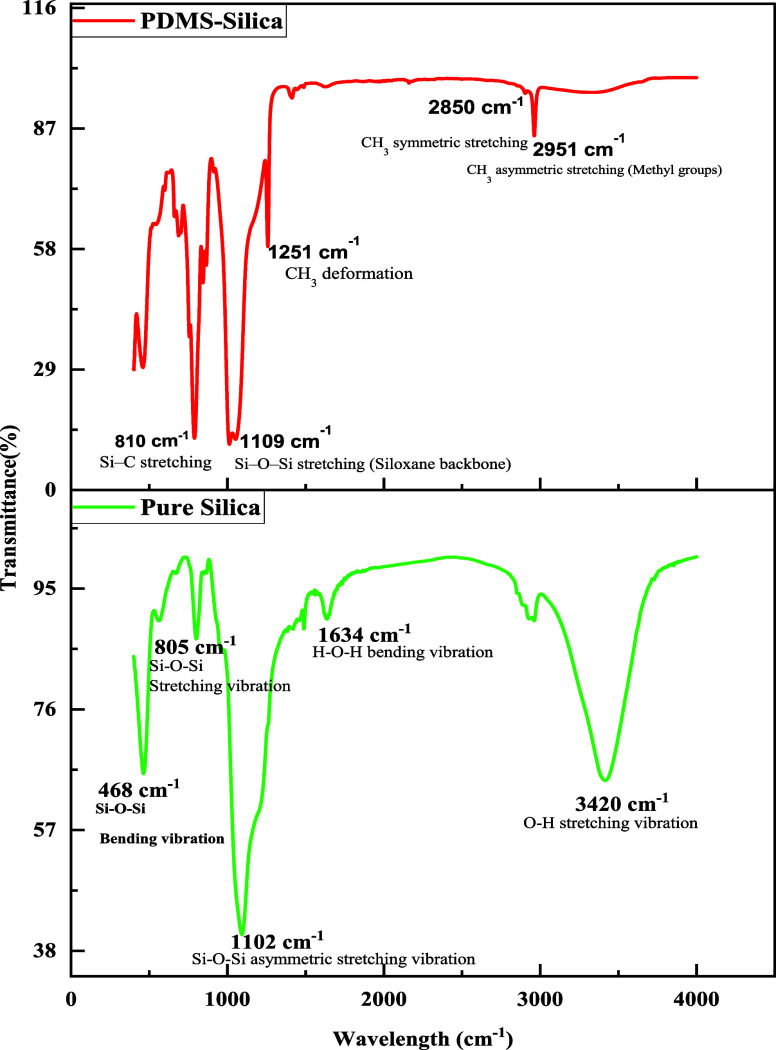
FT-IR stretching plot of poly­(dimethylsiloxane) (PDMS)-silica nanoparticles (SNPs) composite compared with silica, confirming the composite effect between the two components.

### SEM Analysis

The morphological and structural characteristics of the prepared samples were examined by scanning electron microscopy (SEM). As shown in Figure 3(a–c), the SEM images clearly demonstrate the successful embedding of silica nanoparticles (SNPs) within the PDMS matrix. The smooth yet interconnected morphology of the polymeric background indicates that the incorporation of SNPs did not disrupt the bulk integrity of PDMS while at the same time providing localized reinforcement sites. The high-magnification images further reveal that the nanoparticles were well-dispersed, without significant agglomeration, suggesting good compatibility between the SNPs and PDMS. This homogeneous dispersion was essential to ensure consistent interfacial interactions, which directly influence the mechanical and surface properties of the composite material. From Figure 3c, we can see that the mixture had agglomeration, but it did not affect the WCA on the surface. In addition, the corresponding inset images of the SNPs confirm their nanoscale dimensions and spherical morphology, which are preserved after embedding in the polymer network. The observed embedding of SNPs within PDMS can be correlated to improved WCA of the composite. As shown in the representative images in Figure 3(d–f), the larger glass beads are distinctly surrounded by the polymer phase, while the nanoscale silica particles are distributed uniformly throughout the matrix. This multiscale dispersion contributes to a hierarchical structural framework, in which the GBs act as rigid fillers, while the SNPs reinforce the interstitial regions of the polymer. The images confirm that the interface between the GBs, SNPs, and the surrounding matrix is free from significant voids or detachment zones. Such a close arrangement ensures efficient stress transfer across the heterogeneous components, thereby offering improved structural robustness. The embedding of GBs not only provides dimensional stability but also creates primary surface roughness, while the SNPs contribute to secondary roughness. The GBs prevent large-scale deformation under load, while the nanoparticles bridge the polymer–filler interface at a much finer scale, reducing the likelihood of crack propagation.

**Figure 3:**
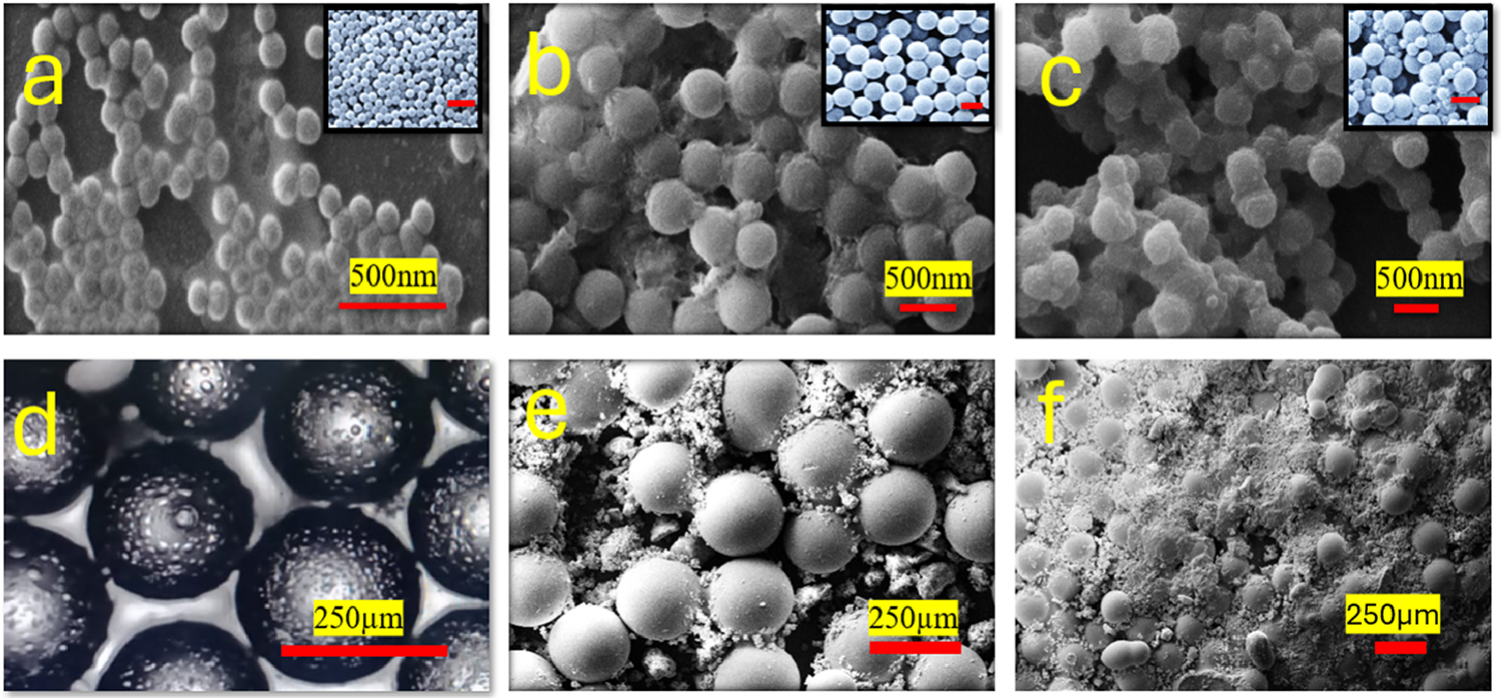
SEM images of fabricated surfaces. (a) 100 nm SNPs embedded onto PDMS. (b) 500 nm SNPs embedded onto PDMS. (c) Mixture of 100 and 500 nm SNPs in a 1:1 weight ratio embedded onto PDMS. Overhead right corner is SEM images of respective SNPs. (d) Optical microscopy image of PDMS-GBs (250 μm). (e) SEM image of PDMS-SNPs (mix)-GBs (250 μm). (f) SEM image of PDMS-SNPs (mix)-GBs (250 μm) with excessive SNPs lowers roughness.

### Water Contact Angle Measurement

Table 2 summarizes the surface compositions, while the corresponding equilibrium water contact angles (WCAs) are presented in Figure 4. Systematic variation of SNPs and the incorporation of GBs result in predictable changes in surface wettability. A maximum WCA of 159° ± 2° was achieved for the PDMS–SNP (mix)–GB (250 μm) composite surface. For surfaces containing SNPs, the use of a dual-size mixture of 100 and 500 nm particles in a 1:1 ratio produced a higher WCA (156° ± 2°) compared to surfaces containing either particle size alone. Based on this observation, further studies were conducted to optimize the PDMS-to-SNP ratio to maximize hydrophobicity. Figure 5a represents the variation of WCA with SNP loading. For surfaces containing only 100 nm (SNP-100) or 500 nm (SNP-500) particles, increasing SNP content led to an initial increase in WCA, followed by a maximum and a subsequent decrease at higher loadings. In contrast, surfaces prepared using the 1:1 mixture of 100 and 500 nm SNPs (SNP-MIX) exhibited a more pronounced enhancement in WCA, reaching a maximum of 156° before decreasing to 140° at higher loadings. The initial increase in WCA is attributed to enhanced nanoscale roughness and improved air entrapment at the solid–liquid interface. However, excessive deposition disrupts the trapped air layer, leading to reduced contact angles. Consistent with the WCA trends, the water sliding angle (WSA) also decreased to a minimum at the optimum SNP loading and increased again at higher loadings (red line in Figure 5a). This behavior arises from reduced contact line pinning at intermediate particle concentrations, which promotes droplet mobility. An optimal hierarchical structure was achieved at an intermediate PDMS-to-SNP ratio of 1:3 for all cases, yielding a stable superhydrophobic surface with a WCA of 156°. Further increase in SNP content (1:4 and 1:5) caused surface nonuniformity, resulting in increased wetting hysteresis and reduced WCA. These trends confirm that dual-scale particle incorporation increased surface roughness, thereby enhancing the apparent contact angle.

**Figure 4:**
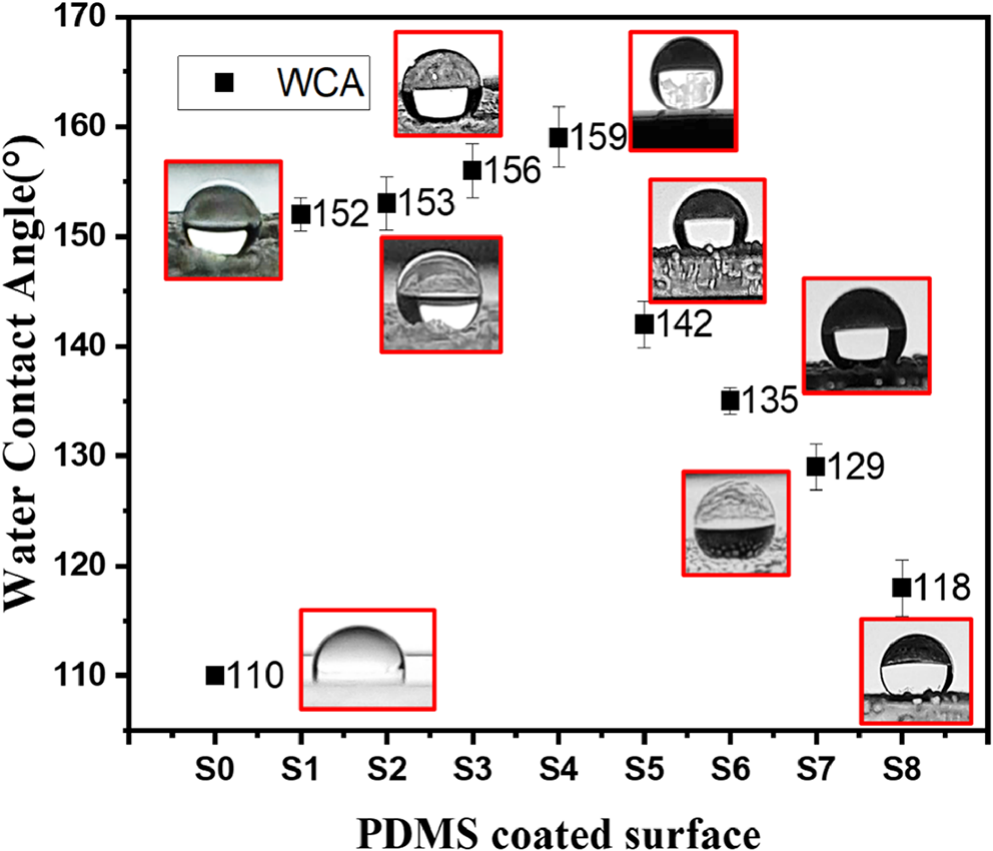
Water contact angle (WCA) analysis of different surfaces prepared using PDMS, SNPs, and GBs of different sizes on a glass substrate, whose description is given in Table 2. Photographic snaps corresponding to the surfaces are posed.

**Figure 5:**
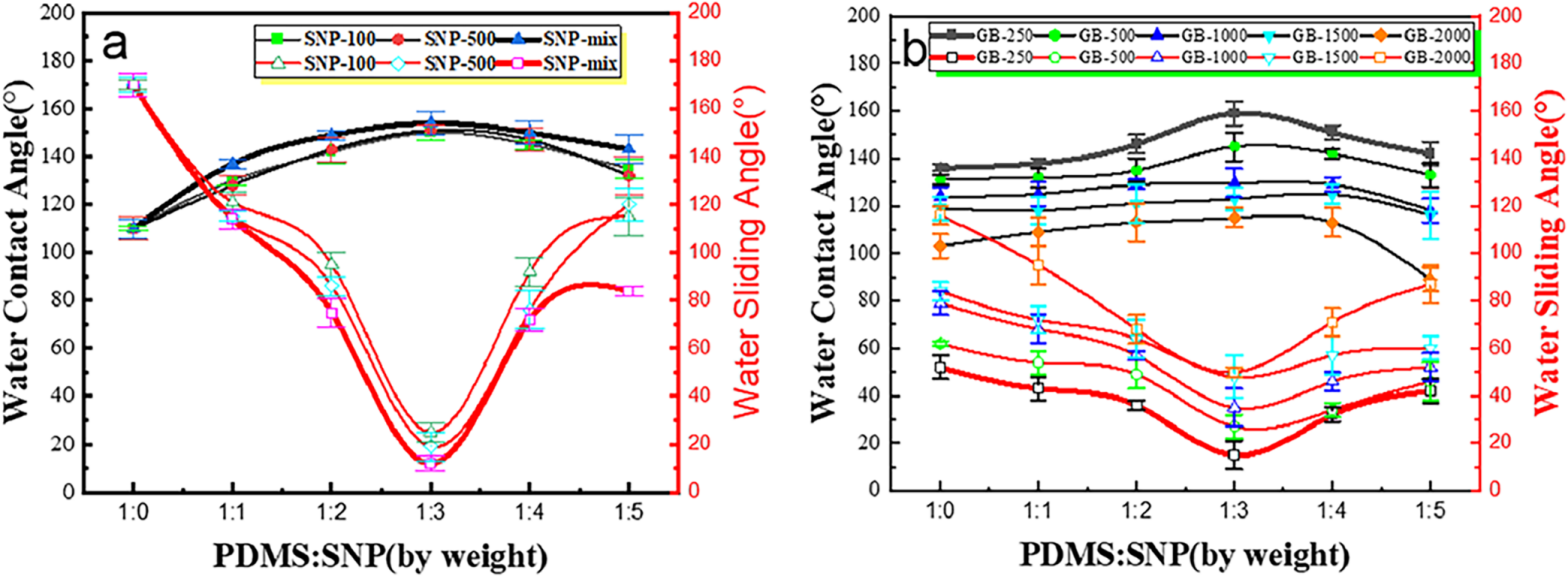
WCA and WSA plots. (a) Effect of embedding dual-sized silica nanoparticles—SNP-100, SNP-500, and their 1:1 mixture (SNP-MIX)—into a PDMS matrix on surface wettability. Water contact angle (WCA) increases, and water sliding angle (WSA) decreases with increasing SNP loading up to an optimum PDMS:SNP ratio of 1:3, beyond which both trends reverse due to reduced effective roughness. The PDMS–SNP-MIX (1:1) composite at 1:3 exhibits the best superhydrophobic performance with a WCA of 156° and a WSA of 10°. (b) Optimization of glass bead (GB) size for single-layer coatings incorporating PDMS–SNP-MIX (1:1) at varying weight ratios. GBs of 250 μm (GB-250) combined with PDMS–SNP-MIX at a 1:3 ratio yield the highest superhydrophobicity, achieving a WCA of 159° and a WSA of 15°, indicating enhanced hierarchical roughness and surface robustness.

**Table 2:** Description of Samples Made for Contact Angle Analysis Given in Figure 5

sample name	description
S0	PDMS
S1	PDMS-SNP (100 nm)
S2	PDMS-SNP (500 nm)
S3	PDMS-SNP (mix) [1:1]
S4	PDMS-SNP (mix)-GB (250 μm)
S5	PDMS-SNP (mix)-GB (500 μm)
S6	PDMS-SNP (mix)-GB (1000 μm)
S7	PDMS-SNP (mix)-GB (1500 μm)
S8	PDMS-SNP (mix)-GB (2000 μm)

After establishing the effect of silica nanoparticles (SNPs) on wettability, we investigated whether adding microscale features could further enhance surface performance. Silica GBs with diameters ranging from 250 to 2000 μm were embedded in PDMS to assess their influence on hydrophobicity. GBs introduce microscale-roughness; however, increasing bead size led to a gradual decrease in WCA (Figure 4 and Table 2). Larger beads increase the wavelength of roughness, allowing the liquid to follow the surface topography more closely, thereby reducing air entrapment and lowering the apparent contact angle. Notably, the combined incorporation of GBs and SNPs introduced hierarchical roughness spanning both micro- and nanoscales, resulting in significantly enhanced hydrophobicity as shown by the black lines in Figure 5b. This dual-scale roughness effectively traps air beneath the droplet. This combination of micro- and nanoscale features is critical for achieving an improved hydrophobic state. The hierarchical roughness induced by the presence of GBs and SNPs served as the principal mechanism governing the observed superhydrophobic characteristics. The corresponding sliding angle data, shown by the red lines in Figure 5b, further confirms that the incorporation of GBs markedly enhanced water mobility, reducing the WSA to as low as 15° for the GB-250. On every surface, similar kinds of superhydrophobic behavior are observed. As shown in Figure 5b, the GB-250 combined with silica nanoparticles (SNP-MIX) exhibited the most pronounced superhydrophobic behavior among all tested samples.

### Relation of WCA with Surface Roughness

To further elucidate the structure–property relationship governing wettability, atomic force microscopy (AFM) was used to quantify the effect of SNP loading on surface roughness for surfaces with and without glass beads (GBs). Representative AFM images are provided in Figure S1 (Supporting Information), and the corresponding relation of root-mean-square (RMS) roughness with WCA is summarized in Figure 6. The results reveal an interesting trend of surface roughness on SNP loading. RMS roughness increases with increasing SNP content and reaches a maximum at a PDMS/SNP (mix) ratio of 1:3, coinciding with the highest measured WCA. It confirms that optimal hierarchical roughness is essential for stabilizing the air entrapment. Beyond the optimum, both RMS roughness and WCA decrease, indicating that excessive SNP loading fills interstitial regions within the PDMS matrix, leading to surface homogenization. A similar trend is observed for PDMS–SNP (mix)–GB (250) composite surfaces, demonstrating that the presence of GBs does not alter the fundamental dependence of wettability on SNP loading but amplifies the roughness at the optimal composition. This observation is further supported by SEM images (Figure 3d–f), which show that excessive SNP deposition reduces surface heterogeneity, thereby diminishing superhydrophobic performance. Hence, achieving an optimum ratio between GBs and SNPs is essential to maintain surface roughness, which collectively governs the performance of the composite.

**Figure 6:**
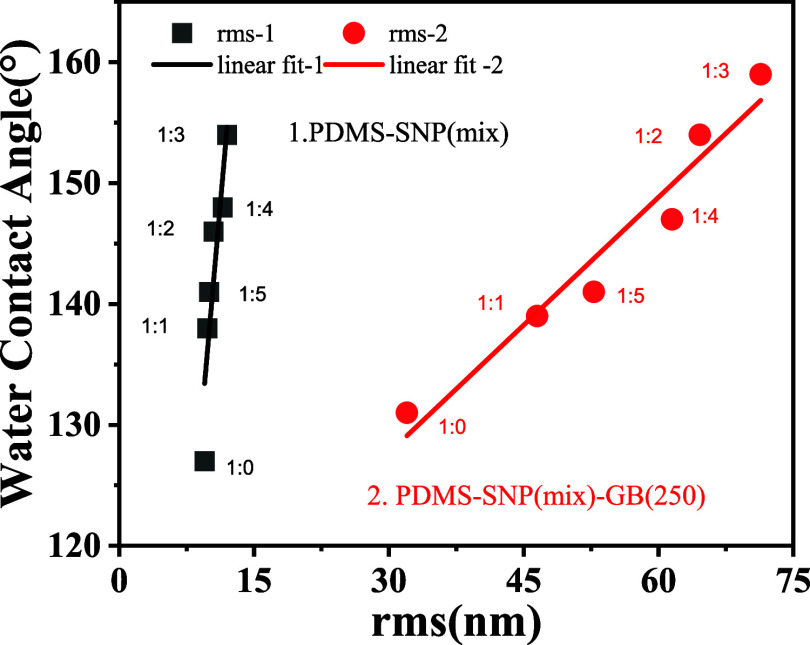
Roughness vs water contact angle. Plot of WCA vs rms for surfaces with and without beads (GBs) where the points represent the weight composition ratio of PDMS and SNP (mix) on the surface.

### Durability Analysis

For assessing the mechanical stability of the surfaces, we performed knife scratch, sandpaper abrasion, and tape peeling tests (see Supporting Information for discussions on sandpaper and tape peeling tests). During the knife scratch test conducted over 40 cycles, both surfaces initially exhibited a gradual decline in WCA and a corresponding increase in WSA, as shown in the plot of Figure 7. Up to 10 cycles, the WCA decreased to 156° and 150° for surfaces with and without GBs, respectively, while the WSA increased from 10° to 12° (with GBs) and from 15° to 17° (without GBs). Around the intermediate stage (∼20 cycles), a pronounced divergence was observed where the surface without GBs (PDMS-SNP (mix)) showed a substantial reduction in WCA and a sharp rise in WSA, leading to a loss of superhydrophobicity, whereas the bead-incorporated surface exhibited minimal change. This behavior highlights the effective anchoring role of the GBs in enhancing surface stability and mechanical robustness. After 40 cycles, significant surface deterioration was evident for the bead-free coating, with the WCA dropping to ∼125°, while the bead-containing surface (PDMS-SNP­(mix)-GB(250)) maintained a WCA close to the superhydrophobic threshold (∼150°). However, increased WSA values were observed at higher cycles, reaching approximately 45° and 20° for surfaces without and with beads, respectively, indicating progressive wear despite the superior durability imparted by the GBs.

**Figure 7:**
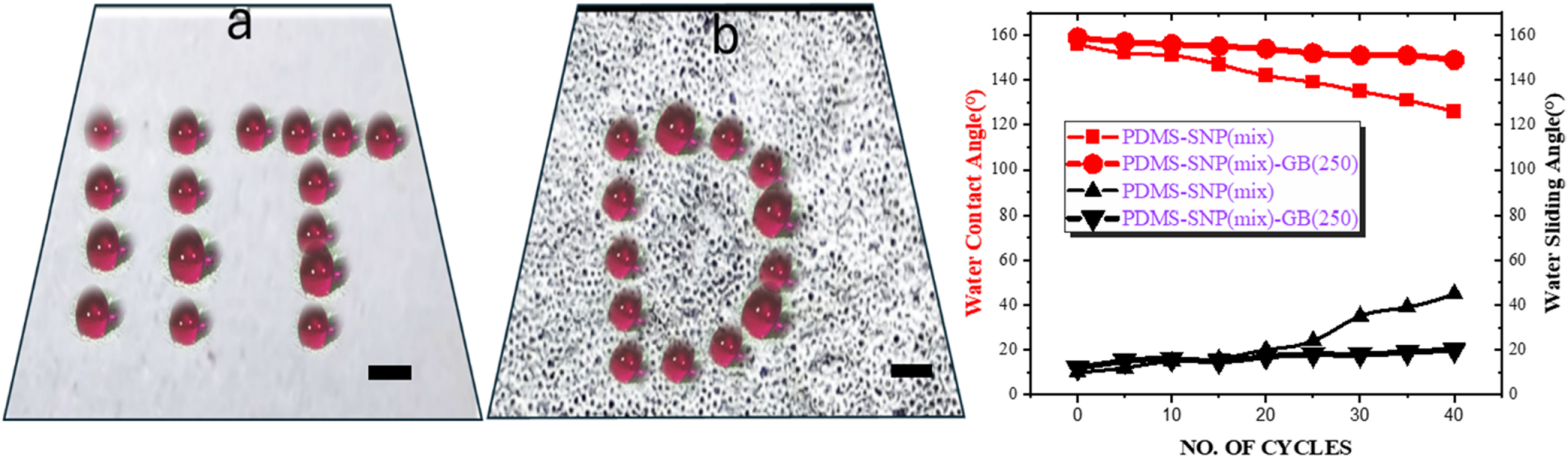
Colored water droplet arranged throughout the surface after the knife scratch test after 40 cycles on both (a) PDMS-SNP (mix) and (b) PDMS-SNP (mix)-GB (250) surfaces, indicating the stability of the surfaces. Scale bar is 1 mm. The plot indicates the gradual decrease in WCA and increases in WSA in both surfaces.

To further assess adhesive strength, we initiated the tape peeling test of the sample without the GBs followed by the test with GBs. As illustrated in Figure S2 of Supporting Information, surfaces with GBs show better stability than the surface without GBs. The sandpaper abrasion test was performed on both surfaces to evaluate their mechanical durability, as illustrated in Figure S3. Consistent with the trend observed in the knife scratch test, the surface containing GBs exhibited higher WCA and lower WSA compared to the surface without beads. The corresponding data are summarized in Table S2 of the Supporting Information for different mesh size sandpapers after 40 cycles of testing. The enhanced durability of GB-reinforced coatings makes them suitable for applications requiring long-term mechanical stability, such as self-cleaning surfaces or industrial hydrophobic coatings. The incorporation of dual-sized SNPs and GBs into the PDMS coating enhances mechanical durability by reinforcing the polymer matrix at both nano- and microscale levels. SNPs improve the cohesive strength of PDMS by restricting polymer chain movement and distributing the applied stress uniformly, thereby reducing crack formation and abrasion damage. Simultaneously, microsized GBs provide structural support and mechanical interlocking with the substrate, improving resistance to peeling and wear. The combined hierarchical architecture also helps preserve the surface roughness responsible for superhydrophobicity, maintaining the coating performance even under mechanical stress. Comparison of mechanical durability strategies in PDMS-based superhydrophobic coatings tabulated in Table S1 highlights SNPs + GBs’ superiority on hard surfaces.

Now we turn our attention to verifying the chemical durability of the surfaces with GBs under different conditions. Initially, the stability was tested on multiple substrates, including wood, metal, plastics, and concrete. As shown in Figure 8a, the water contact angle (WCA) consistently remained above 150°, demonstrating excellent substrate-independent superhydrophobicity. Furthermore, the coating retained its high WCA even after exposure to acidic and basic solutions for more than 30 min (Figure 8b), with negligible variation observed across a wide pH range. This confirms that the coating exhibits chemical stability in both acidic and alkaline environments. The stability of these surfaces can be attributed to the hydrophobic methyl groups in PDMS, which effectively repel polar solvents, including those at extreme pH, thereby preserving the water-repellent nature of the surface. The thermal stability of the PDMS–SNP (mix)–GB (250) coating was further evaluated through hot-water exposure tests. As shown in Figure 8c, the WCA decreased slightly until 156.5° after immersion in boiling water, extending the temperature up to 120 °C. This stability was due to the inherent thermal resistance of PDMS, whose Si–O–Si backbone remains intact within a wide temperature range (−60 to 260 °C).^46^ The nonpolar methyl groups in PDMS continue to repel water molecules, ensuring persistent surface hydrophobicity under thermal stress.^47^ Additionally, the embedded GB–SNP network preserves the micro/nanoscale roughness essential for superhydrophobic behavior, as both glass beads and SNPs exhibit excellent thermal stability well above the temperature of boiling water. But there is a decline in WCA to 152.5° on the extended duration of boiling water, indicating swelling behavior of PDMS. Hot water (∼100 °C) lowers its surface tension from ∼72 mN/m (25 °C) to ∼59 mN/m, enabling easier penetration into surface roughness, collapsing air pockets, and wetting the underlying texture. Longer durations amplify diffusion of uncured PDMS oligomers or water vapor into the matrix, restructuring low-energy methyl groups and partially hydrolyzing siloxane bonds, increasing surface energy (Figure 8d).

**Figure 8:**
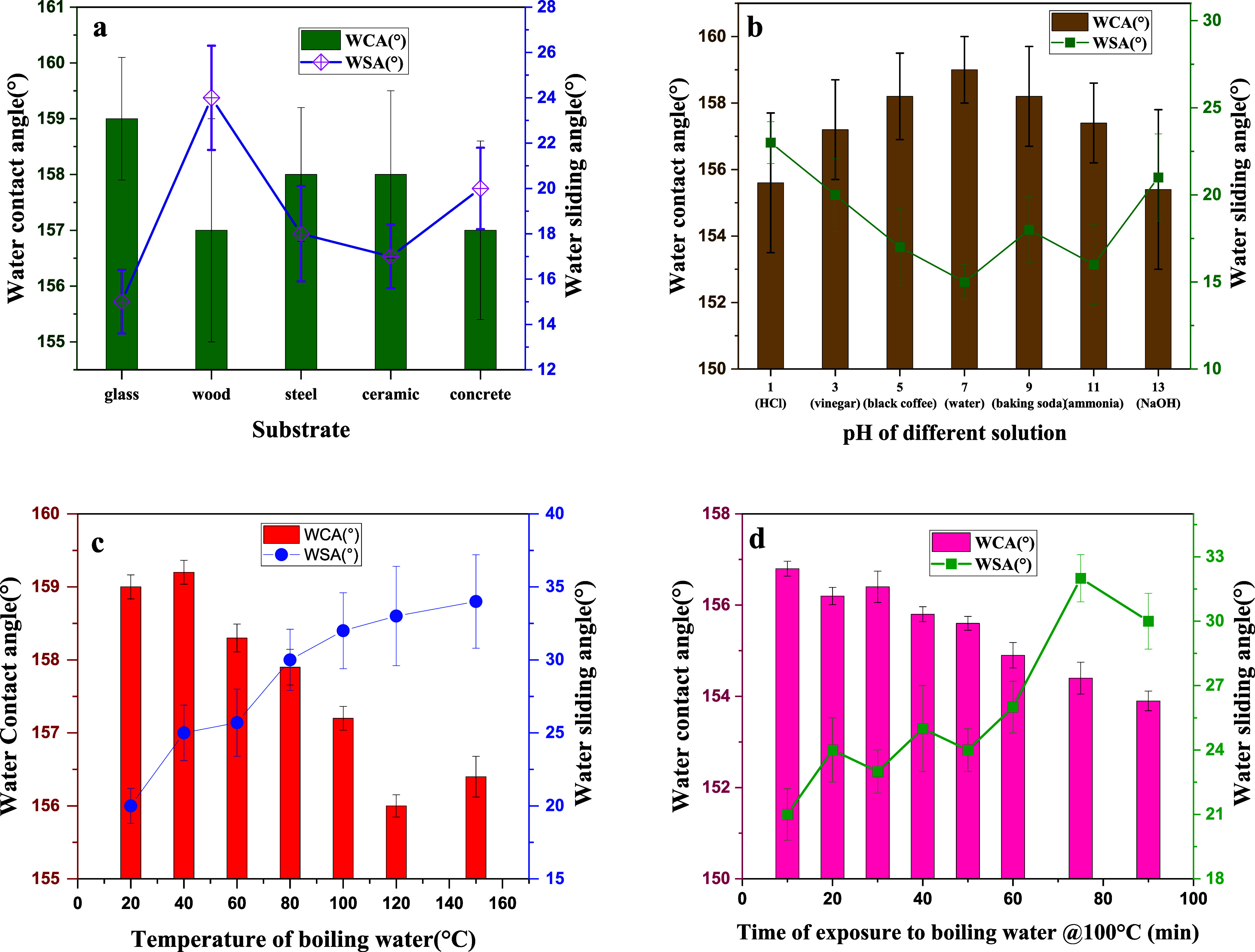
Chemical durability test of PDMS-SNP (mix)-GB (250) surface. (a) WCA of coating on different substrates. (b) WCA of coating on glass dipped under different acid/alkaline solutions. (c) WCA when exposed to hot water. (d) WCA when exposed to boiling water at different times.

## Conclusion

In this work, we have demonstrated superhydrophobic behavior of silica nanoparticles (SNPs) embedded in PDMS-coated surfaces. The incorporation of SNPs increases the apparent contact angles by enhancing surface roughness. Increasing the particle size leads to higher effective roughness, resulting in higher contact angle and lower roll-off angles. A further increase in water contact angle is observed for coatings containing mixed SNPs. Notably, the combined incorporation of SNPs and glass beads (GBs) yields water contact angles of 159°. Integration of GBs significantly improves the mechanical and chemical durability of the coatings. The bead-reinforced surfaces exhibit enhanced resistance to scratching, abrasion, and chemical exposure. Beads act as structural reinforcements, protecting the hierarchical micro- and nanoscale roughness and helping to preserve the hydrophobic state even after mechanical wear. Although coatings without beads exhibit good initial performance, their rapid degradation under mechanical stress limits their practical applicability. In contrast, bead-reinforced coatings demonstrate sustained water repellence and durability, making them suitable for applications in harsh environments. Further optimization of GBs size, distribution, and integration techniques could enhance the performance of these coatings, paving the way for robust, cost-effective superhydrophobic materials for a wide range of applications.

## Supplementary Material







## Notes

Plastron effect of coated sample when dipped in water bath (MP4)
Jumping droplet on coated sample (MP4)
AFM images, mechanical stability test (tape peeling test and sandpaper abrasion test) (PDF)
