# Supplementary material for: Hierarchically Reinforced PDMS–Silica Coatings for Durable Superhydrophobicity
Source: ACS Omega. 2026 Jul 3;11(28):41926–35. doi: 10.1021/acsomega.6c01949 (PMC13393187; doi:10.1021/acsomega.6c01949)
Supplement: Supplementary file 3 [file ao6c01949_si_003.pdf]

Supplementary Information for:

Hierarchically reinforced PDMS–Silica coatings for durable superhydrophobicity

*Agneyarka Mohapatra, Somnath Ghosh**

Department of Chemical Engineering, Indian Institute of Technology, Delhi, New Delhi, India, 110016

*Correspondence: somnath@iitd.ac.in

AFM analysis

The atomic force microscopy (AFM) observations were carried out in tapping mode to check the topography and roughness of the surface. The root-mean square (RMS) roughness value was found from area 80 μm× 80 μm figure by using the Gwyddion software[1], [2].

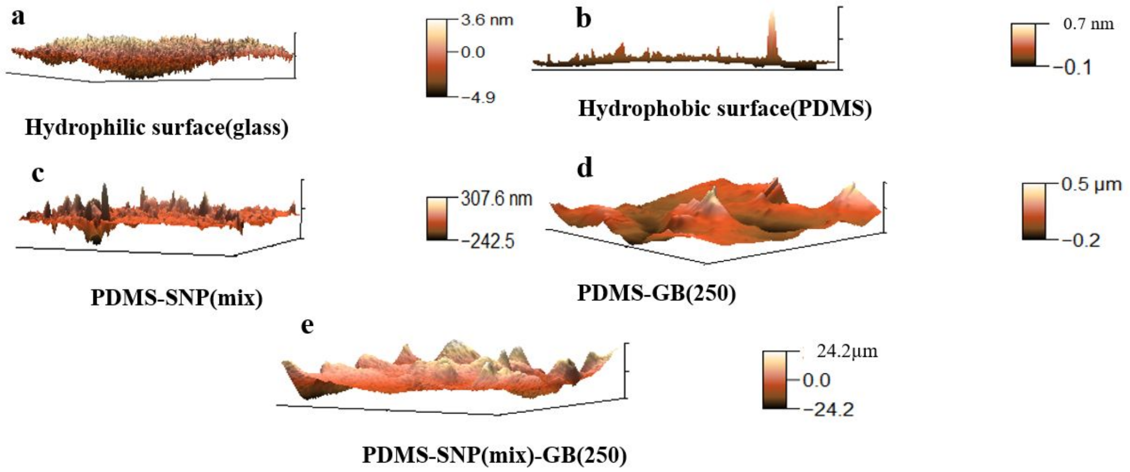

Fig.S1. ***AFM analysis of critical surfaces****. (a)hydrophilic glass surface with 1.11nm roughness value (RMS). (b)Hydrophobic PDMS coated glass with roughness around 3.12nm. (c)Superhydrophobic PDMS-SNP (mix) [1:1] on glass surface offers 18.56nm roughness value. (d) Hydrophobic PDMS-GB (250μm) with 56.4nm roughness. (e) Superhydrophobic PDMS-SNP (mix) [1:1]-GB (250μm) surface with 78.2nm roughness. Area of analysis was 80 X 80 μm²*.

Atomic Force Microscopy (AFM) was employed to quantify the topographical evolution of surfaces fabricated with sequential modification at respective steps. All AFM scans were performed over an 80 × 80 µm² micro area. The evaluated values of root mean square (RMS) roughness demonstrated a systematic increase corresponding to the transition from hydrophilic to hydrophobic and ultimately superhydrophobic surfaces, supporting the direct influence of hierarchical micro–nano structuring on wetting behaviour. The bare hydrophilic glass substrate in Fig S1(a) exhibited an RMS roughness of 1.11 nm, characteristic of a quiet smooth and uniform surface. Upon coating with PDMS in Fig. S1(b), the surface roughness increased to 3.12 nm, indicating formation of a thin elastomeric layer with slight topographical undulations. Although the roughness change was modest, PDMS inherently reduces surface energy, which explains the hydrophobic behaviour observed (WSA=110°) despite limited nano-scale texturing. A significant shift occurred when PDMS was blended with silica nanoparticles (SNPs) in a 1:1 ratio and deposited onto glass substrate. This superhydrophobic PDMS–SNP in Fig.S1(c) surface showed an RMS roughness of 18.56 nm, confirming that incorporation of rigid nanoparticles generated micro–nano protrusions essential for air-pocket entrapment[3], where heterogeneous surface morphology reduces the solid–liquid contact fraction leading to increase in contact angle to 153°. Further upon introduction of glass beads (GBs of 250 µm) into PDMS in Fig.S1(d) resulted in a much higher roughness of 56.4 nm. The increase stems from the microscale curvature and surface asperities created by partially embedded GBs, producing mechanical heterogeneity that enhances hydrophobicity even without SNPs slightly around 125°.Thereafter with highest roughness, the hybrid superhydrophobic PDMS–SNP (1:1)–GB (250 µm) in Fig.S1(e) composite surface exhibited an RMS roughness of 78.2 nm. This hierarchical combination of micron-sized GBs and SNPs produced multi-length-scale texturing, closely mimicking natural superhydrophobic structures such as lotus leaves. The amplified surface roughness supports maximum air-trapping capability and minimizes liquid adhesion, explaining the superior water repellence observed for this architecture with WCA around 159°.

Mechanical stability test

The mechanical and chemical stability of the surfaces were evaluated through a series of tests.

Adhesion strength and wettability were examined using a tape-peeling test following ASTM D3330 standard. Surface stability and robustness test were conducted using knife scratch test[4] in accordance with ASTM D6677. Duct tape was repeatedly applied and peeled off the coated surfaces, and the WCA and WSA were measured after every 10 peeling cycles to monitor changes in wettability[2]. We performed sandpaper abrasion test[5] to assess the mechanical robustness of coatings in accordance with ASTM D3884. The coated substrates were placed face-down on sandpapers of varying grit sizes (no mesh,800, 1000, 1500, and 2000 mesh), with a 100 g weight applied to ensure uniform load. The surfaces were abraded back and forth for 10cm 40 cycles along the ruler placed parallel to the sandpaper[6].

**Strategy**	**Durability Mechanism**	**Limitations vs. SiO2+GB+PDMS**	**Ref.**
Single NPs in PDMS	Filler stiffening; nano-roughness	Low macro-scale toughness; cracks under abrasion (e.g., 5-50m sandpaper)	Hydrophobic SiO_2/PDMS[7]; Silica (100/500nm) /PDMS[8]
PDMS + epoxy/plasticizer	Chemical crosslinking	Brittle failure; loses hydrophobicity faster	ER@SiO_2@PDMS[9]; shellac/PDMS[10]
Hierarchical wrinkles/pillars	Dual-scale roughness (micro PDMS + nano etch)	Substrate-dependent; poor scratch resistance without hard fillers	DLC/PDMS[11]; etched PDMS [12]
Quartz@SiO2/PDMS	Hierarchical quartz core-shell	Good wear/weather but no micro-bead interlocking	Quartz-reinforced[13]

Table S1. Comparison of mechanical durability strategies in PDMS-based superhydrophobic coatings.

Tape peeling test

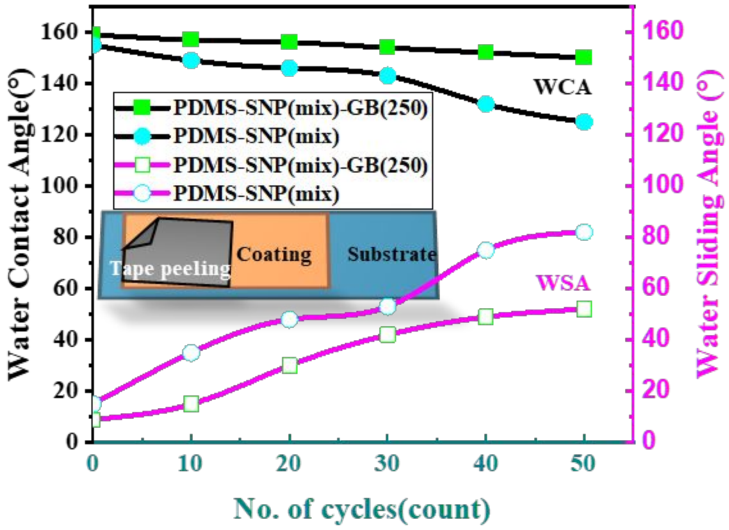

Fig.S2. Plot and experimental schematic of number of tape peeling cycles vs WCA and WSA with and without beads(GB).

The tape-peeling test, as illustrated in Fig. S2, further demonstrates the robustness of the surface. Like sand abrasion test here too we encountered the samples with and without beads to study the contact angles. The coating exhibited an initial WCA of 156° and a sliding angle of 18°, indicating excellent superhydrophobicity. As per the plot after 5 cycles of peel offs, the WCA dropped to 150°, and the WSA increased to 35°, showing moderate loss of surface roughness for the coating without glass beads. After 30 Cycles, the WCA decreased significantly to 142°, and the WSA rose to 50°, suggesting detachment of SNP (mix) and deformation of the PDMS matrix. After 50 Cycles, the WCA was reduced to 125°, and the coating exhibited partial hydrophobicity, transitioning closer to towards more wetting. On the other hand, coatings with beads (GBs) provide better stability of surface. It displayed a similar initial performance, with a WCA of 159° and a sliding angle of 10°. After 5 Cycles, The WCA reduced slightly to 157°, and the sliding angle rises to 15°, demonstrating minimal damage. After 30 cycles, the WCA dropped to 155°, and the WSA increased to 38°, with the hierarchical roughness largely intact. After 50 Cycles, the WCA remained above 151°, and the sliding angle stayed under 45°, indicating superior mechanical stability. The beads provided structural reinforcement, protecting the SNPs and PDMS matrix from detachment and deformation. The GBs also distributed the peeling force more evenly across the surface, reducing localized damage. The enhanced durability of GBs-reinforced coatings makes them suitable for applications requiring long-term mechanical stability.

Sandpaper abrasion test

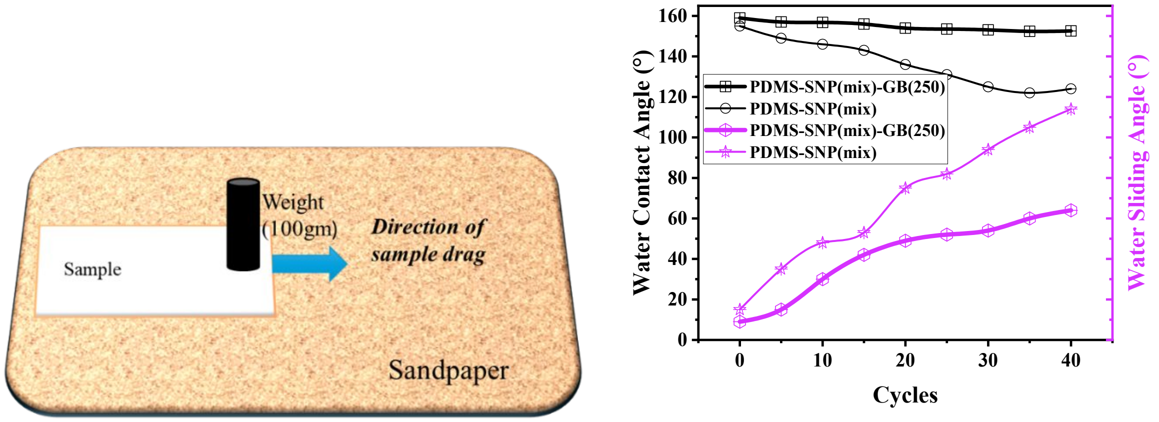

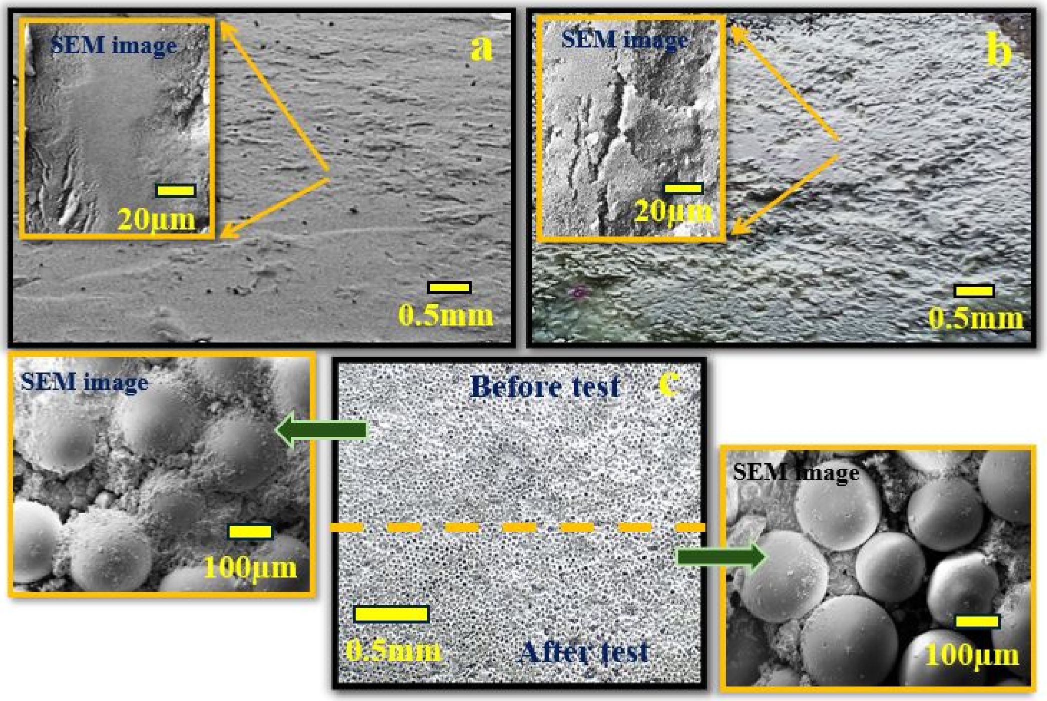

Fig.S3. Experimental schematic of sandpaper abrasion test and WCA-WSA plot vs 40 times test results with 2000 mesh sandpaper with comparison with and without GBs on PDMS-SNP (mix). PDMS-SNP (mix) surface (a) before test and (b)after test with morphology from corresponding SEM image. (c) PDMS-SNP (mix)-GB (250) surfaces with before and after test morphology.

**Sandpaper roughness**	**PDMS-SNP (mix)**		**PDMS-SNP (mix)-GB (250)**	
	**WCA (°)**	**WSA (°)**	**WCA (°)**	**WSA (°)**
No mesh	153 ±2	20 ±2	158 ±4	10 ±1
800 mesh	112 ±4	No rolling	148 ±2	95 ±2
1000mesh	115 ±5	135 ±4	150 ±4	84 ±3
1500 mesh	120 ±4	129 ±5	151 ±3	76 ±4
2000 mesh	124 ±2	114 ±4	152 ±5	64 ±2

Table.S2. Tabulated data of WCA-WSA on surfaces after sandpaper abrasion test of both PDMS-SNP (mix) and PDMS-SNP (mix)-GB (250) upon 40th cycle sandpaper test.

For the sandpaper abrasion tests, samples were subjected sequentially to sandpapers of decreasing roughness, progressing from 800 mesh (coarse) to 2000 mesh (fine), and finally to a smooth surface. This protocol was applied to coatings both with and without glass beads. Figure S3 schematically illustrates the abrasion procedure, with particular emphasis on the evaluation of water contact angle (WCA) and water sliding angle (WSA) after abrasion using 2000-mesh sandpaper to assess surface uniformity. The abrasion test was performed over a sliding distance of 10 cm for 40 cycles. The durability data obtained after the 40th abrasion cycle using different sandpaper meshes for PDMS–SNP (mix) and PDMS–SNP (mix)–GB (250) coatings are summarized in Table S1. Upon analysing the plot in Fig S3 for 2000mesh sandpaper, the WCA of PDMS-SNP (mix) decreases from 156° to 124° due to large scale erosion of SNP (mix) from the surface. Along with high increase of WSA angles from 20° to 114° due to low adherence to the surface. Morphology of corresponding surface supports the claim in Fig.S3(a)-(b). While in the case of PDMS-SNP (mix)-GB (250) had downward slope with shallow decrease from 158° to 152° retaining the superhydrophobic characters to longer cycles of abrasion. The cause of decrease to about 7° can be due to erosion of lightly embedded SNP (mix). However, a comparatively sharp increase in WSA from 10° to 64° was observed, indicating enhanced surface adhesion due to the removal of loosely embedded SNP (mix) during abrasion clearly shown in Fig.S3(c). Following this trend, abrasion tests were also conducted using 800, 1000, and 1500 mesh sandpapers. As the sandpaper mesh progressed from coarse to fine, the comparative WCA–WSA data consistently exhibited a decrease in WCA accompanied by a corresponding increase

in WSA, in agreement with the above interpretation tabulated in Table S1. A comparative robustness analysis between coatings with and without glass beads clearly demonstrates the superior abrasion resistance of the PDMS–SNP(mix)–GB(250) coating relative to the PDMS–SNP (mix) system. These results highlight the critical role of incorporating mechanically robust fillers such as glass beads for applications demanding high wear resistance, including self-cleaning surfaces and outdoor protective coatings.

References

[1] A. Lazauskas, D. Jucius, L. Puodžiukynas, A. Guobienė, and V. Grigaliūnas, "SiO2-Based Nanostructured Superhydrophobic Film with High Optical Transmittance," *Coatings*, vol. 10, no. 10, p. 934, Oct. 2020, doi: 10.3390/coatings10100934.

[2] S. Afrin, D. Fox, and L. Zhai, "Organic superhydrophobic coatings with mechanical and chemical robustness," *MRS Commun.*, vol. 10, no. 2, pp. 346–352, Jun. 2020, doi: 10.1557/mrc.2020.33.

[3] H. Y. Erbil and C. E. Cansoy, "Range of Applicability of the Wenzel and Cassie−Baxter Equations for Superhydrophobic Surfaces," *Langmuir*, vol. 25, no. 24, pp. 14135–14145, Dec. 2009, doi: 10.1021/la902098a.

[4] Q. Luo *et al.*, "Recent Advances in Multifunctional Mechanical–Chemical Superhydrophobic Materials," *Front. Bioeng. Biotechnol.*, vol. 10, Jul. 2022, doi: 10.3389/fbioe.2022.947327.

[5] R. S. Sutar *et al.*, "Superhydrophobic PVC/SiO2 Coating for Self-Cleaning Application," *Macromol. Symp.*, vol. 393, no. 1, p. 2000034, 2020, doi: 10.1002/masy.202000034.

[6] Y. Lu, S. Sathasivam, J. Song, C. R. Crick, C. J. Carmalt, and I. P. Parkin, "Robust self-cleaning surfaces that function when exposed to either air or oil," *Science*, vol. 347, no. 6226, pp. 1132–1135, Mar. 2015, doi: 10.1126/science.aaa0946.

[7] S. Vlassov *et al.*, "Adhesion and Mechanical Properties of PDMS-Based Materials Probed with AFM: A Review," *Rev. Adv. Mater. Sci.*, vol. 56, no. 1, pp. 62–78, May 2018, doi: 10.1515/rams-2018-0038.

[8] Q. Ke, W. Fu, H. Jin, L. Zhang, T. Tang, and J. Zhang, "Fabrication of mechanically robust superhydrophobic surfaces based on silica micro-nanoparticles and polydimethylsiloxane," *Surf. Coat. Technol.*, vol. 205, no. 21, pp. 4910–4914, Aug. 2011, doi: 10.1016/j.surfcoat.2011.04.073.

[9] L. Zhou *et al.*, "Durable ER@SiO2@PDMS superhydrophobic composite designed by double crosslinking strategy for efficient oil-water separation," *Polymer*, vol. 245, p. 124722, Apr. 2022, doi: 10.1016/j.polymer.2022.124722.

[10] R. Soni *et al.*, "Scalable and Durable Superhydrophobic Coating Using Shellac-Based Bioadhesive and Hierarchical Silica Nanoparticles," *Langmuir*, vol. 41, no. 5, pp. 3269–3277, Feb. 2025, doi: 10.1021/acs.langmuir.4c04180.

[11] Y. Rahmawan, M.-W. Moon, K.-S. Kim, K.-R. Lee, and K.-Y. Suh, "Wrinkled, Dual-Scale Structures of Diamond-Like Carbon (DLC) for Superhydrophobicity," *Langmuir*, vol. 26, no. 1, pp. 484–491, Jan. 2010, doi: 10.1021/la902129k.

[12] B. Cortese, S. D'Amone, M. Manca, I. Viola, R. Cingolani, and G. Gigli, "Superhydrophobicity Due to the Hierarchical Scale Roughness of PDMS Surfaces," *Langmuir*, vol. 24, no. 6, pp. 2712–2718, Mar. 2008, doi: 10.1021/la702764x.

[13] H. Zhang *et al.*, "High-durability polydimethylsiloxane/quartz@SiO2 superhydrophobic coating with enhanced mechanical robustness and wear resistance," *Polym. Test.*, vol. 152, p. 108967, Nov. 2025, doi: 10.1016/j.polymertesting.2025.108967.
